# **Structure-Based** Design of Ricin Inhibitors

**DOI:** 10.3390/toxins3101233

**Published:** 2011-10-13

**Authors:** Karl Jasheway, Jeffrey Pruet, Eric V. Anslyn, Jon D. Robertus

**Affiliations:** Department of Chemistry and Biochemistry, University of Texas, Austin, TX 78712, USA; Emails: karl.jasheway@utexas.edu (K.J.); jpruet@mail.utexas.edu (J.P.); anslyn@austin.utexas.edu (E.V.A.)

**Keywords:** ricin, structure-based drug design, pteroic acid, RTA, pterin chemistry, ribosome inactivating protein, differential scanning fluorimetry, virtual drug screening, ICM

## Abstract

Ricin is a potent cytotoxin easily purified in large quantities. It presents a significant public health concern due to its potential use as a bioterrorism agent. For this reason, extensive efforts have been underway to develop antidotes against this deadly poison. The catalytic A subunit of the heterodimeric toxin has been biochemically and structurally well characterized, and is an attractive target for structure-based drug design. Aided by computer docking simulations, several ricin toxin A chain (RTA) inhibitors have been identified; the most promising leads belonging to the pterin family. Development of these lead compounds into potent drug candidates is a challenging prospect for numerous reasons, including poor solubility of pterins, the large and highly polar secondary binding pocket of RTA, as well as the enzyme’s near perfect catalytic efficiency and tight binding affinity for its natural substrate, the eukaryotic ribosome. To date, the most potent RTA inhibitors developed using this approach are only modest inhibitors with apparent IC_50_ values in the 10^−4^ M range, leaving significant room for improvement. This review highlights the variety of techniques routinely employed in structure-based drug design projects, as well as the challenges faced in the design of RTA inhibitors.

## 1. Introduction

Ricin, from the castor plant *Ricinus communis*, is a type II holotoxin belonging to the Ribosome Inactivating Protein (RIP) superfamily [1,2]. Type II RIPs are comprised of a catalytic A subunit, and a lectin B subunit which mediates cellular uptake. For ricin, these chains are referred to as ricin toxin A chain (RTA) and ricin toxin B chain (RTB), respectively. Type I RIPs consist of only the catalytic subunit. Type I RIPs appear to play a role in plant antiviral defenses; they are not cytotoxic unless they can be delivered to the cytoplasm, for example by breaching the cell [3].

Ricin has received significant attention since the infamous umbrella tip assassination of Georgi Markov publically demonstrated the extreme lethality of the toxin [4,5]. Due to its ease of extraction in large quantities from castor beans, which are processed worldwide on an industrial scale, there is a real threat of ricin being used as a biological warfare agent. It is therefore important to develop an antidote for the deadly toxin as a defense against such an attack.

The use of structure-based drug design is an attractive approach for the development of small molecule inhibitors for the treatment of ricin intoxication. The use of X-ray crystallography and/or NMR spectroscopy to obtain structural information detailing the interaction between an inhibitor and its target macromolecule is the cornerstone of structure-based drug design. The X-ray structure of ricin is known [6,7,8], and complexes with substrate analogs have revealed key features of the RTA active site [9,10]. When the macromolecular target structure is known, medicinal chemists can rationally develop synthetic derivatives of an existing inhibitor to improve potency by creating more favorable binding interactions with the target. This review focuses on the use of this approach in the development of inhibitors targeting the catalytic A subunit of ricin, highlighting progress made in this endeavor as well as obstacles that remain to be overcome.

## 2. Ricin Structure and Action: Implications for Inhibitor Design

### 2.1. X-Ray Structure of Ricin

The X-ray structure of the ricin holotoxin was initially solved to 2.8 Å resolution [7] and later refined at 2.5 Å [8], allowing the molecular description of the individual protein chains [8,11]. The cloned A chain was later crystallized and solved in two different space groups at 2.1 Å resolution [12] and 1.8 Å respectively [13]. The X-ray structures allow an analysis of the suitability of each chain as a drug design target.

### 2.2. RTB Is Not a Good Prospect for Structure-Based Inhibitor Design

RTB might seem like the logical target for inhibitor design. If small molecules could be made that would bind tightly to it and preclude cell uptake, that would be ideal. The analysis of the X-ray structure showed that the B chain of ricin is composed of two related domains, which are each composed of three related subdomains. Only one subdomain of each domain binds galactosides, and these two binding sites are over 50 Å apart, on opposite ends of the protein [14,15,16]. The binding sites individually exhibit only weak binding to galactosides [17] with *K*_d_ values in the millimolar range. This weak binding at each site is biologically tolerable because the two widely separated sites contribute independently to the free energy of binding, and because the target cell surface is literally covered with galactosides [18]. This is not useful for inhibitor design, however. The RTB galactose binding pockets are small (120-150 A^3^ as calculated by Q-Sitefinder [19]), and make only weak interactions with galactose [20,21]. Designing effective ligands to the shallow, polar galactose sites is difficult, and the two sites are also too far apart for a small molecule to bind both sites simultaneously. In contrast, RTA has two larger pockets that are within close proximity to each other, making it possible for a molecule with two fragments connected by a linker to be designed to fit both pockets at once. This makes RTA the more attractive target for structure-based drug design and justifies our focus on it for this review.

### 2.3. RTA Is a Plausible, but Challenging Inhibitor Design Target

Ricin Toxin A chain chemically inactivates the eukaryotic ribosome by hydrolysis of a single adenine base (A^4324^) on the sarcin-ricin loop (SRL) of the 28S rRNA of the large subunit [20,21]. Ricin shows a *K*_m_ for ribosomes around 1 μM, and a *k*_cat_ of around 1500 min^−1^, depending on the ribosome species [12,21,22]. The catalytic efficiency of this hydrolysis reaction, *k*_cat_/*K*_m_, is near the diffusion limit. This means that ricin has evolved to enzymatic perfection for this specific ribosome inhibiting reaction. In contrast, ricin attacks naked RNA at a rate about 10^4^-10^5^ times more slowly [21], and only at nonphysiological pH [23], suggesting this activity is essentially a nonspecific side reaction of its biological function [14].

**Figure 1:**
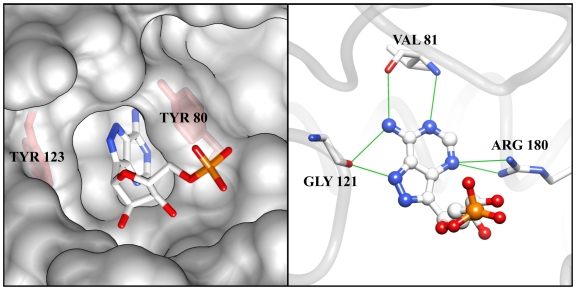
Structure of RTA complexed with substrate analog, formycin 5'-monophosphate (PDB ID: 1FMP): The binding of the AMP analog formycin 5'-monophosphate to the active site of RTA is shown below. The aromatic ring analogous to the adenine base of the natural substrate is sandwiched between Tyrosine 80 and 123 (**left**), and makes six hydrogen bonds, shown as solid green lines (**right**), within the active site.

The micromolar *K*_m_ for ribosomes is indicative of the tight binding affinity that RTA has for its natural substrate. It is useful in structure-based inhibitor design to understand the chemical nature of that binding. The heart of substrate binding is the accommodation of the target adenine base in a “specificity” pocket in the RTA active site. The nature of this interaction was observed in a complex with formycin monophosphate (FMP), a non hydrolyzable analog of AMP [9]. Crystallographic studies of RTA showed that in the absence of substrate, the RTA specificity pocket was “closed”; that is, the side chain of Tyr 80 rotated to block its entrance [15]. However, in the presence of a substrate analog, RTA adopts an “open” conformation in which Tyrosine 80 moves to accommodate the substrate, forming a π-stacking network with the adenine base and Tyrosine 123 (Figure 1). In addition to the π-stacking interactions, the substrate forms six hydrogen bonds with the binding pocket, conferring specificity for the adenine base. Successful design of potent inhibitors of RTA is expected to require that both the π stacking and hydrogen bonding interactions be retained.

The active site of RTA can be described as having two binding pockets when it is in the open conformation: the primary adenine specificity pocket and a slightly larger secondary pocket. These two pockets are separated by the side chain of Tyrosine 80. The second pocket was proposed, based on model building, to accommodate a guanine base from the invariant GAGA ribosomal target sequence [9]; this has been confirmed by the X-ray structure of an RTA complex with a locked cyclic nucleotide [10]. The guanine base forms an aromatic stack with Tyrosine 80, and thereby forms an extended stack of Tyr 123, the adenine in the specificity pocket, Tyr 80 and the guanine base. However, the binding of guanine appears to be weak as we have been unable to soak the free base, nucleoside or nucleotide into that site. Its observed binding in the cyclic tetranucleotide is speculated to be driven by the conformational rigidity of that ligand which reduces configurational entropy of binding. Our efforts to construct small dinucleotide substrate analogs that bind to both pockets have been unsuccessful, illustrating the importance of conformational rigidity required for occupation of the second pocket.

**Figure 2:**
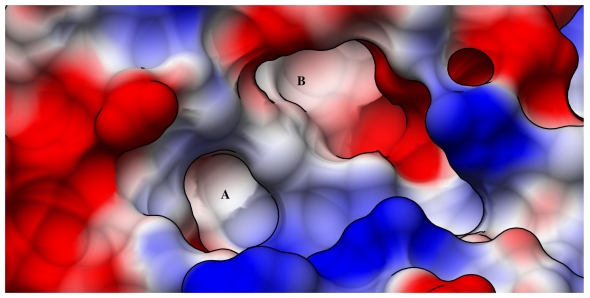
Electrostatic potential surface map of RTA: RTA is shown in the open conformation, with the adenine specificity pocket labeled (**A**) and the secondary binding pocket labeled (**B**) Surfaces with positive electrostatic potential are shaded blue while those with negative potential are shaded red.

Despite the aromatic stacking interactions described above, the two RTA pockets and the surrounding surfaces are largely polar, as shown in Figure 2. This is not surprising given that the natural substrate is the ribosome. However, it makes it necessary for inhibitor platforms to conform to stringent polarity restraints in order to precisely complement the binding site and optimize binding interactions. Such complementary compounds would have polar surface areas which generally incur a high penalty in desolvation energy upon binding. This issue has historically caused difficulty in implementing structure-based drug design for targets with large polar binding sites [16,24]. In RTA, the area between the two pockets contains several positively charged arginine residues that accommodate the phosphate backbone of the natural substrate RNA. This must also be taken into consideration when synthetically optimizing inhibitors.

## 3. Ricin Inhibitors

Recently, ricin inhibitors have been identified through classical high throughput screening strategies using large libraries of compounds to protect cultured cells from intoxication [25]. However, ricin intoxication is a complex process involving cell uptake, trafficking to the ER, release to the cytoplasm and ribosome inactivation. The cell based assay does not identify which process or protein [26] an anti-ricin compound is acting on. Another cell-based screen identified an anti-ricin compound that acts, not by inhibiting RTA action, but by disrupting cell trafficking [27]. Without a clear understanding of the macromolecular target being inhibited, it is difficult to rationally improve upon the initial high throughput hits. Of those inhibitors identified in the cell-based assay, only a small percentage showed anti-RTA activity in cell-free systems. For now, we will focus on those inhibitors known to act on RTA.

**Figure 3:**
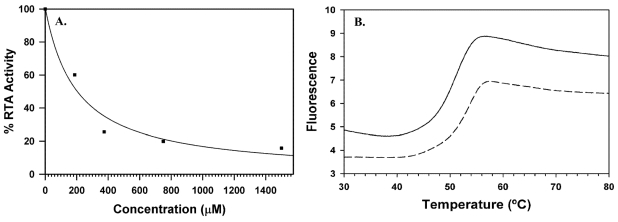
RTA dose response and melting curves: (**A**) The dose response for *N*-(2-(phenylamino)ethyl)-7-carbamoyl pterin (compound **12**) is shown, as measured in the cell-free translation assay. Individual data points, shown as dots were fit via least squares regression to a hyperbolic decay function; (**B**) The melting curve for compound **12** is shown as measured by DSF. The melting curve for RTA alone is represented by a solid line, and the curve for RTA in the presence of compound **12** is represented by a dashed line.

To date, compounds that inhibit RTA action have been mainly discovered by virtual screening and structure-based design [28,29,30,31,32]. Assaying potential inhibitor compounds for anti-RTA activity has proven to be a difficult task. Our current assay uses a cell-free translation system to produce firefly luciferase, with luminescence as the reporter for measuring the decrease in translation activity due to RTA and rescue of translation caused by RTA inhibitors. An example of a dose response curve is shown in Figure 3a.

Differential scanning fluorimetry (DSF) is utilized as a complementary technique to our cell free translation assay to provide further evidence of compounds binding to RTA. The technique relies on a fluorescent dye which becomes active upon binding to the hydrophobic surface patches of RTA that are exposed as the protein denatures under a controlled temperature gradient. This allows for the melting temperature of RTA to be accurately measured in the absence and presence of potential ligands using a real-time PCR instrument. The observation of an increase in melting temperature induced by the ligand indicates tight binding to the protein and stabilization of the folded form [33,34]. Figure 3b shows an example set of melting curves in which a significant thermal shift was observed. Only compounds identified by virtual screening have shown positive results in DSF, which is consistent with what we have observed with the translation assay.

Since RTA interacts with rRNA, it might seem that RNA based inhibitors could be useful anti-ricin agents. Indeed, Schramm and his coworkers have created a number of tightly binding RNA analogs to RTA [35]. However, RNA based inhibitors, although mechanistically interesting, are not expected to be useful drugs since they are biochemically labile and have difficulties crossing cell membranes.

Other small molecule inhibitors of RTA have been identified using computer simulated docking of large virtual compound libraries to the open form of RTA and confirming their anti-RTA activity using cell-free translation assays [29,30,31]. Many of these were successfully soaked or co-crystallized with RTA, and their X-ray structures revealed binding to the adenine specificity pocket. The inhibitors that yielded complex structures had numerous structural similarities, the most important of which being an exocyclic amine that donates two hydrogen bonds to the backbone carbonyls of Valine 81 and Glycine 121. Another important characteristic is aromaticity, which is necessary for the stacking interaction with the two tyrosine side chains. 

**Figure 4:**
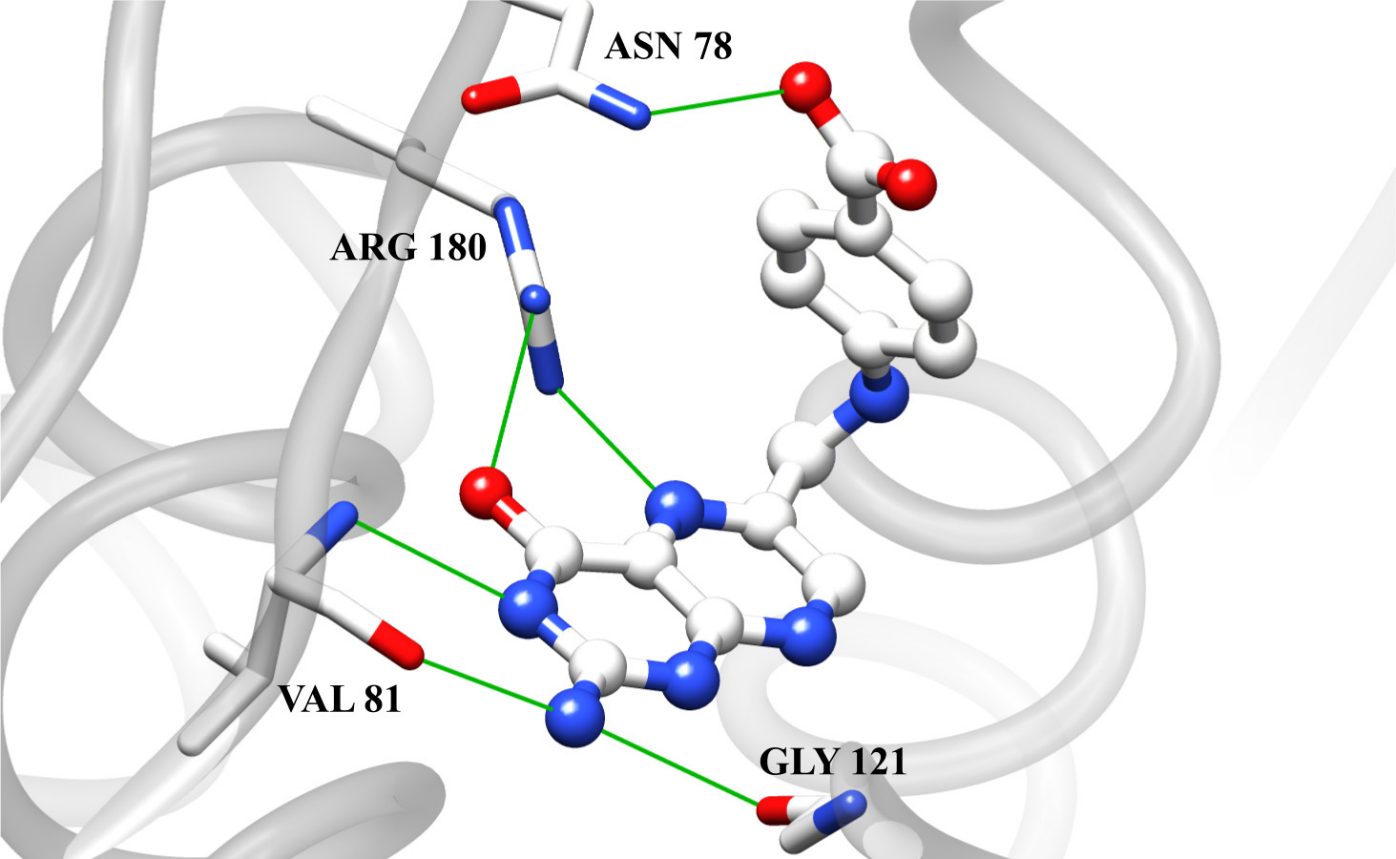
Pteroic Acid in complex with RTA: The crystal structure of the complex of RTA with pteroic acid is shown (PDB ID: 1BR6). Hydrogen bonds are depicted as solid green lines.

The first RTA inhibitor identified from virtual screening was pteroic acid, PTA, which had an apparent IC_50_ of 600 μM [31]. The crystal structure of the RTA-pteroic acid complex, shown in Figure 4, reveals that the pterin group binds in the adenine specificity pocket, making six hydrogen bonds, and that the benzoic acid moiety is in close proximity to the secondary pocket. Unfortunately, efforts to improve the inhibitory activity of pteroic acid by attaching pendants at the benzoic acid group were unsuccessful due to synthetic restrictions.

The clearest way to improve inhibition of RTA is to design a compound that occupies both active site binding pockets: the specificity pocket that holds the substrate adenine and the second pocket. The second pocket, being larger, more open, and more polar than the primary pocket, presents exceptional difficulties in identifying or designing compounds with specific affinity for this site. For this reason, a fragment-based approach would be impractical. Our approach is to use the scaffolds known to bind the specificity pocket with high affinity and synthesize extensions, which possess both rigidity and complementarity to the region between the two binding pockets. Rigidity means that the linker should have a minimum of entropically unfavorable rotatable bonds that greatly impede ligand binding [32]. These extensions will be developed into linkers to which aromatic groups can be attached. The position and conformational rigidity of the linker will have to be a strong enough driving force for the attached aromatic pendant to be forced into the second pocket, where it could make stacking interactions with Tyrosine 80.

Scaffolds that have been shown to bind with reasonable affinity to the specificity pocket include adenine, guanine, pterin, and dihydroxyamino-pyrimidine [29,30,31]. We have explored synthetic derivatization of each of these platforms. Each has its own synthetic difficulties that need to be overcome to generate novel inhibitors. Once novel compounds are made, each must be evaluated as an RTA inhibitor. Currently, the most promising platform is the pterin series. Despite solubility issues both in synthesis and in application, the pterin-based inhibitors show the most reproducibility in both the luciferase kinetic assay and X-ray crystallographic studies. The other scaffolds have been less successful and have therefore been abandoned. In this report we will focus on novel synthesis of pterin-based RTA inhibitors.

## 4. Novel Compound Synthesis

Based on the structural data for pterin binding in the specificity pocket, it is apparent that synthetic extensions can be made from pterin at positions 6 and 7, which could, in principle, reach toward the second pocket on the enzyme surface; most naturally occurring pterins such as pteroic acid, neopterin, and folate are substituted at the 6 position [36,37,38,39]. As part of our program we needed to explore derivatization at both positions.

**Scheme 1:**
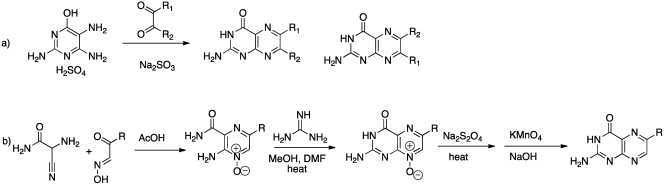
Pterin synthesis by Isay and Taylor methods.

A serious drawback of pterin chemistry comes from their notorious insolubility; the complementary hydrogen bond donors and acceptors within pterin result in solute-solute interactions too strong for most solvents to overcome. There are two main methods by which pterin rings are constructed: Isay condensation and the Taylor method, illustrated in Scheme 1. Isay condensation, the more straightforward method, involves condensation of a dicarbonyl compound with 6-hydroxy-2,4,5-triaminopyrimidine to provide the pterin product Scheme 1a [40]. With symmetric dicarbonyl compounds, only one product is formed; but unsymmetrical dicarbonyls result in two regioisomers, 6 and 7 [36]. For this reason, Taylor and coworkers developed an alternative, step-wise construction of the pterin core Scheme 1b [38,41].

**Figure 5:**
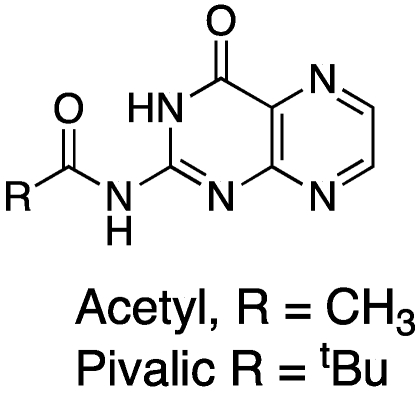
Protected pterins for improved solubility.

Regardless of the method used in the construction of the pterin, solubility problems must be addressed. One of the most common methods of improving pterin solubility is through protection of the N2-exocylic amine as an amide (Figure 5); we have used pivalic-protected pterins to greatly improve solubility in such common organic solvents as ethanol, ethyl acetate and dichloromethane [42]. Unfortunately, we have often observed this pivalic group to be too labile for many of the reactions we desire to make, with the deprotected pterin precipitating out of solution prior to product formation.

**Scheme 2:**
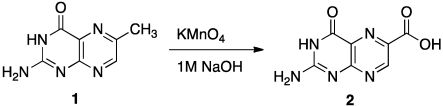
Derivitizing 6-methyl pterin.

Once we identified a useful N2 blocking group, we began a program to derivatize pterin at both the 6 and 7 positions. We created a 6-methyl pterin that could be oxidized to generate 6-carboxy pterin, 6CP, as shown in Scheme 2 [43].

**Scheme 3:**
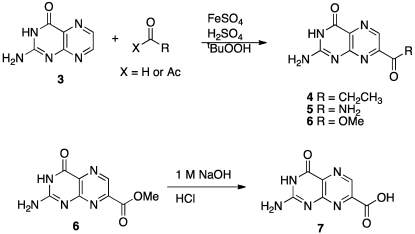
Synthesis of various 7-substituted pterins.

The Minisci group has developed radical-species methods that allow insertion into heterocyclic rings, which we explored for pterin [44,45,46]. Previous studies suggested this reaction would be regiospecific in pterins and would provide the 7-isomer, rather than the more common 6-substituted pterin [47]. The synthesis of 7-carboxy pterin, 7CP, is shown in Scheme 3.

We purified and tested both 6CP and 7CP as RTA inhibitors. 6CP showed little, if any, measureable inhibitory activity, and soaking experiments showed it did not bind to RTA crystals. In contrast, 7CP was a relatively potent inhibitor, with an IC_50_ of 240 μM, and showed strong binding to the crystals [48]. It is possible that other, uncharged modifications at the 6-position could produce useful inhibitors; but with limited synthetic resources, we decided to pursue pterin compounds modified at the 7-position.

**Scheme 4:**
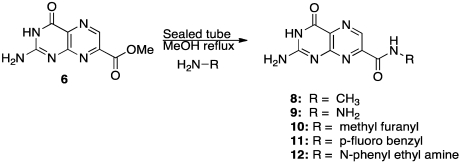
Synthesis of amide derivatives.

One of our first efforts focused on creating amide derivatives of 7CP. Due to solubility restrictions, we opted for a route illustrated in Scheme 4, suspending the methyl ester of 7CP in methanol with a large excess of the amine and heating in a sealed tube overnight. This method was successful in providing us with the amides, in yields ranging from 59-92% [48]. A variety of pendants have been attached in this fashion. These have been tested for RTA inhibition and binding examined by X-ray diffraction. A highlight of the pterin results are summarized in Table 1. Many of the compounds are less soluble than the charged 7CP, and many have a lower affinity. This indicates the pendants are not making specific interaction with RTA, which is confirmed by X-ray crystallography. In many cases the pterin ring is firmly bound in the specificity site, but the pendant tail appears disordered. 

**Table 1:** Summary of pterin-based inhibitors.

**Entry**	**Structure**	**Name**	IC_50_ *	**Resolution**
PTA	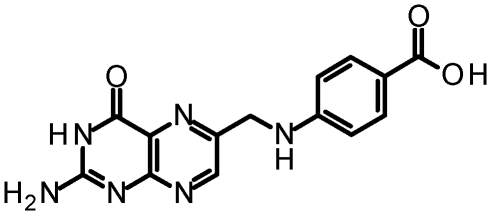	Pteroic acid	600 μM	2.30 Å
1	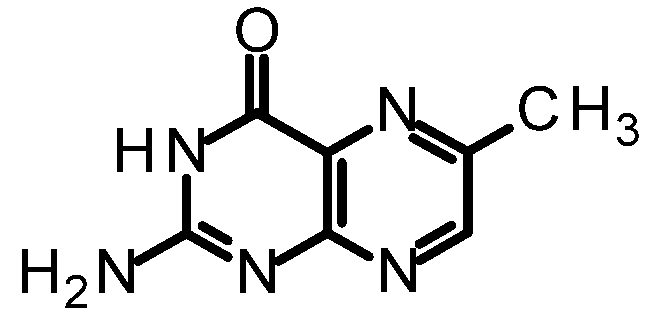	6-methyl pterin	No Inhibition	NA
2	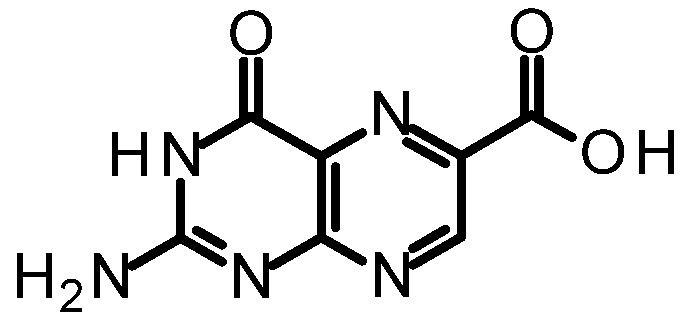	6-carboxy pterin	No Inhibition	NA
7	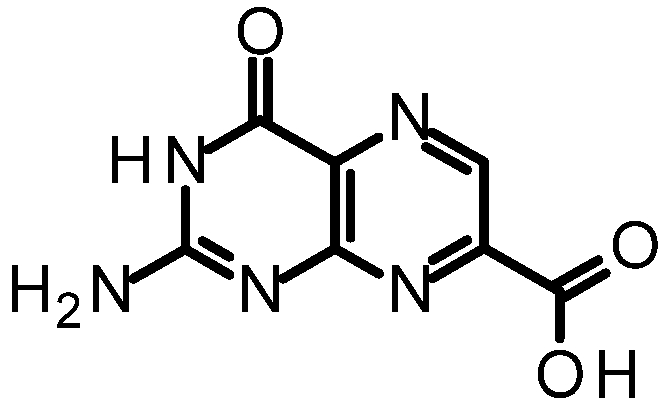	7-carboxy pterin (7CP)	240 μM	1.29 Å
5	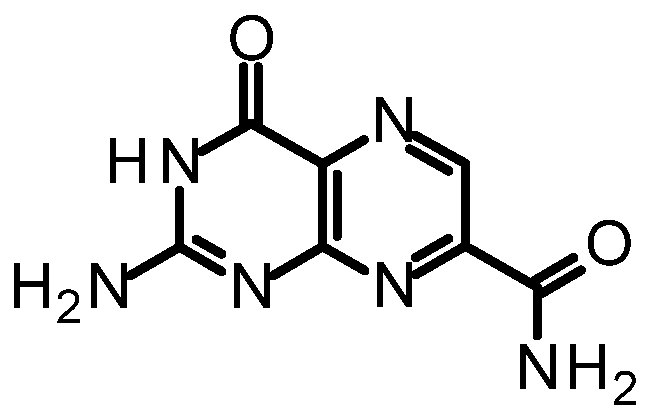	7-carbamoyl pterin	No Inhibition	1.75 Å
8	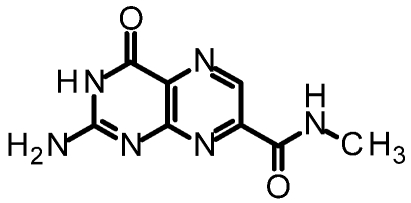	*N*-methyl-7-carbamoyl pterin	1.6 mM	1.26 Å
9	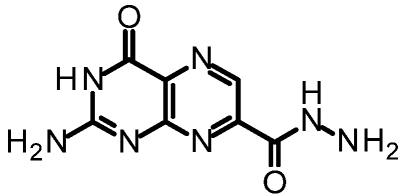	7-hydrazide pterin	500 μM (35%)	1.97 Å
4	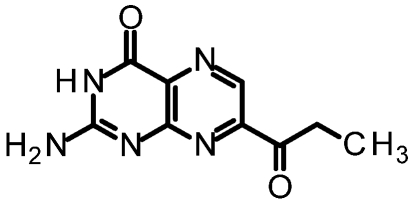	7-propionyl pterin	750 μM	1.35 Å
10	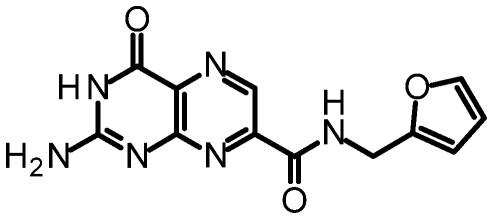	*N*-(furanylmethyl)-7-carbamoyl pterin	380 μM	1.89 Å
11	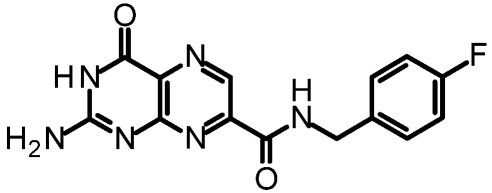	*N*-(4-fluorobenzyl)-7-carbamoyl pterin	570 μM	NA
12	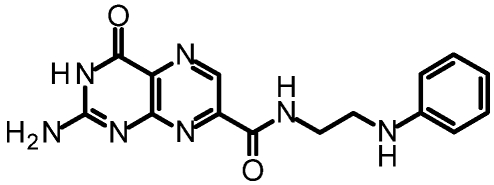	*N*-(2-(phenylamino) ethyl)-7-carbamoyl pterin	200 μM	1.75 Å

* When 50% inhibition was not reached; % inhibition is given in parenthesis at maximum concentration.

## 5. Plans for Inhibitor Optimization

**Figure 6:**
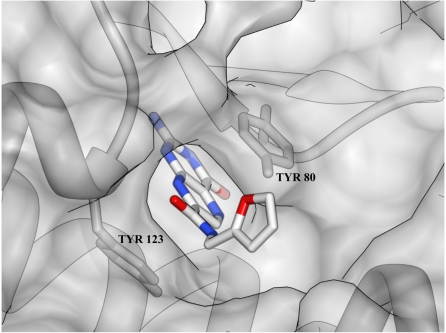
Complex of RTA and Compound **10**: The crystal structure of *N*-(furanylmethyl)-7-carbamoyl pterin in complex with RTA reveals that the furanylmethyl moiety is oriented favorably for further development as a linker extending into the second pocket beyond TYR 80. The distance between the furan and the hydroxyl group of TYR 80 is measured at 4.2 Å, which is too long for a hydrogen bond.

Future work with the pterins will be aimed at extending the 7CP linkers with chemical groups that can bind into the second pocket. These pendant groups should ideally be able to make specific polar contacts with protein residues. Efforts will initially be focused on derivatizing primary amines as linkers. Two linkers of particular interest are the aminomethyl furan from compound **10** and the ethylene diamine from compound **12**. Based on the crystal structure of compound **10**, the furan group, although not close enough to TYR 80 to form a hydrogen bond, is oriented in a favorable position such that derivatization of the 5 position would allow the attachment of pendants extending into the secondary pocket (Figure 6). The ethylene diamine linker can be reacted with a variety of activated acids, esters, nitriles, and isocyanates to yield amide, guanidinium, and urea linkages. The high hydrogen bonding capacity of these groups should allow the linker to make favorable contacts with the highly polar surface between the two binding pockets, promoting conformational stability.

A number of docking programs are available that can predict the interactions of small molecules with protein surfaces [49,50,51]. These can be useful, not only for virtual screening of large libraries [52,53], but also in designing derivatives of known inhibitors. Programs of this type explore many conformations of the ligand within the binding cavity, and compute the energies of interaction for each test conformation. Our experience in a limited number of cases suggests that these programs predict ligand binding conformation with reasonable accuracy [32,34].

**Figure 7:**
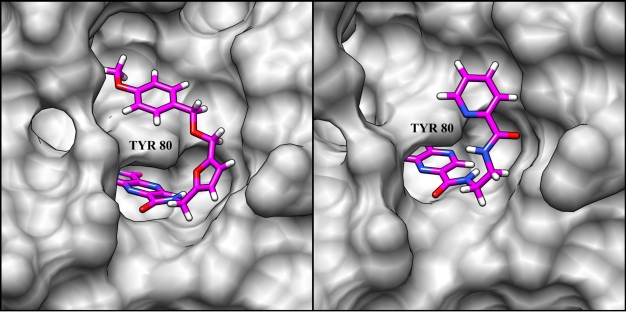
Docking results from ICM simulation: Predicted binding poses of pterin derivatives bearing furan (**left**), and ethylene diamine (**right**) linkers are shown. Hydrogen bonds (not shown) are predicted to form between TYR 80 and the ether group in the compound on the left, and between TYR 80 and the pyridine group in the compound on the right.

Through the use of the virtual docking program ICM [54], the binding of theoretical compounds can be predicted prior to them being synthesized. By docking potential pterin derivatives, synthetic efforts can be focused on those compounds that showed the most promising results, avoiding those who performed poorly. Synthesis of new pterin derivatives bearing the furan and ethylene diamine linkers will be guided in such a way. The predicted binding of two theoretical pterin derivatives are shown in Figure 7. Both designed molecules are predicted to bind with higher affinities than are their parent compounds, which are known inhibitors (Table 1).

In addition to conformational rigidity, specific interactions between the linker and the protein are crucial for the compound to make an energetically unfavorable 180° degree turn to reach the secondary pocket. The prediction of binding orientation via virtual docking is helpful in identifying linkers capable of making such interactions. However the validity of these predictions has yet to be demonstrated. While a compound may be capable of binding to RTA in such a way that the linker makes specific interactions with RTA, the energy of those interactions must be significant enough to force the molecule into a conformation necessary to make them. Once a linker is confirmed via X-ray crystallography to fulfill these requirements, virtual docking can then be used to identify pendants that would make optimal interactions with the second pocket. By achieving occupancy of the second pocket, the anti-ricin activity of these compounds is expected to improve dramatically. Therefore, the immediate obstacle that remains to be overcome in the design of potential ricin antidotes is the synthesis of a linker that possesses both rigidity and specificity for RTA. 
